# Medical Opinion and Movement

**Published:** 1911-02-18

**Authors:** 


### Medical Opinion and Movement.

### Tuberculin and Syphilis.

In a communication to the Société Medicale des Hôpitaux, Dr. Siredey reported the results of subcutaneous injections of tuberculin in eleven cases of syphilis. Of these eleven only one was known to be tuberculous and one was suspected, but nine gave a positive reaction to the injection—that is to say, had a temperature above 38.5° C. for some hours afterwards. The author has obtained similar results with the cuti- and with the intradermo-reaction, so that according to these observations it is impossible by any tuberculin reaction to distinguish tuberculous from syphilitic lesions. Only the presence of the bacilli of Koch and a positive inoculation of guinea pigs with presence of the bacilli in the lesions afford a definite proof of the tuberculous nature of a lesion at the present time.

### Otitis Media.

In the *Medizinische Klinik*, Dr. S. Blum draws attention to a sign which he considers of considerable clinical and diagnostic value in cases of otitis media. If the finger be placed under the angle of the inferior maxilla in the hollow between this bone and the sterno-mastoid muscle and a little pressure be applied, pain will always be produced in cases of acute otitis media of the same side. The pressure exercised must only be of a moderate degree, as energetic pressure will always cause pain even in normal conditions. One must also be able to exclude the presence of a cervical abscess, an adenopathy, or a cutaneous inflammation. The author points out that this sign is of especial service in very young children or babies who are unable to explain their own feelings and with whom examination with the speculum is often difficult by reason of the narrowness of the external meatus and also on account of the restlessness of the child under examination. Moreover, the speculum may reveal nothing abnormal in appearance even in presence of a purulent otitis. Dr. Blum cites a case of a baby aged five months suffering for twenty-four hours from pain, crying, refusing the breast, with fever and rapid pulse. Examination of the chest and abdomen showed nothing abnormal. Applying pressure between the angle of the lower jaw and the sterno-mastoid muscle at once made the child cry, draw its head away, and push away the offending finger. Traction on the ear, however, caused no pain. Otoscopic examination by a specialist showed nothing abnormal, and he accordingly refused to perform paracentesis, as advocated by Dr. Blum. Two days later the membrane perforated spontaneously, with an abundant purulent discharge. The author claims to have verified this sign in more than two hundred cases of acute otitis media.

### Anaphylaxis.

At a meeting of the Société de Biologie, Drs. Lesné and Dreyfus gave an account of some research work carried out by them on dogs and rabbits to determine the effect of the gastric and pancreatic secretions upon anaphylactic substances. They used actino-congestine for the purpose, which was introduced directly into the stomach, small intestine and large intestine respectively, and also injected intravenously. They found that no anaphylactic effect results after introduction into the stomach or small intestine, but introduced into the large intestine the effect is the same as when injected intravenously and was followed by the death of the animal. By further experiments they found that the hydrochloric acid of the gastric juice does not prevent the production of anaphylaxis with actino-congestine, but if these substances are acted upon by pepsin or pancreatin for some time in an incubator, subsequent intravenous injection shows a marked attenuation of its anaphylactic property, so that five or ten times the dose is required to produce the same effect. Similar experiments were carried out with the white of eggs on fowls. Much the same results were obtained, but the ferments in this case completely abolish the anaphylactic effects of the white of egg.

### Chronic Appendicitis.

At a meeting of the Société Medicale des Hôpitaux an interesting discussion took place upon the occurrence of conditions of chronic appendicitis simulating tuberculosis. The discussion was opened by Dr. Faisans, who maintained that not infrequently patients consult a medical man on account of general symptoms of ill-health—loss of weight and of strength, lassitude, thoracic pains and pains in the back and the right side, and are treated for pulmonary tuberculosis, whereas actually they are suffering from some chronic inflammatory condition of the appendix. There is frequently a slight rise of temperature in the evening, which is increased by walking or hard work, as in the case of tuberculosis. The condition may last for months or years without any marked change taking place. There may be some abnormal sounds at the right base, resulting from a pleuro-pneumonic infection at the time of the acute attack of appendicitis some time previously; or some diminution of vesicular murmur at the right apex, actually of no significance, but sufficient to justify the diagnosis in view of the general symptoms. Careful inquiry into the previous history of the patient may disclose facts relative to an acute attack of appendicitis in the past, and examination of the abdomen may confirm the diagnosis by a characteristic tenderness over McBurney's point. Several others testified to the truth of Dr. Faisans' views, and cited cases identical in all respects to those portrayed by him. Dr. Siredey pointed out that in young women with dysmenorrhœa chronic appendicitis may be accompanied by a sclero-cystic degeneration of the ovaries. In such cases the affected ovary should be removed as well as the appendix. Dr. Thiroloix gave an account of three cases of pulmonary tuberculosis accompanied by chronic appendicitis, which he found to be also of a tubercular nature, and he was inclined to the view that many such cases of chronic appendicitis, accompanied by general symptoms, slight feverishness, are of a like kind.

### Small-pox in Ancient Egypt.

An interesting historical discovery is reported in the *Journal of Pathology and Bacteriology* by Drs. Armand Ruffer and Ferguson. This is nothing less than a mummy whose skin still shows what are apparently clear evidences of severe small-pox. There was found a bullous or vesicular eruption which in form and distribution bore a striking resemblance to that of small-pox, so these two observers removed a piece of the skin and, after softening it, prepared it for miscroscopical examination. In the sections thus prepared, it is interesting to note, bacilli are easily demonstrable by the ordinary modern methods of staining; and though the authors admit that probably most of these multiplied and flourished greatly between the actual decease and the mummification, they do not think that any later date than that of the latter event can be assigned to them. The epidermis is completely gone from the specimen, but dome-shaped vesicular spaces can be demonstrated in the prickle cell layer of the dermis, wherein small-pox lesions originate nowadays. In one or two of the sections the authors identified traces of the vertical septa which sub-divide the developing vesicle of variola. It cannot be said that they have proved that this mummy actually had small-pox, but at least they have shown a fair possibility of it. The body was that of a tall man of middle-age, and came from one of the royal tombs.

### Cordite Eating.

In Lieut.-Col. R. J. S. Simpson's "Medical History of the South African War," now appearing in the *Journal of the Royal Army Medical Corps*, is given the result of a special inquiry instituted into a new drug-habit which first came to light during that campaign. Owing to the dearth of matches, the men got into the habit of using the little rods of cordite with which their cartridges were loaded, in order to light their tobacco. The peculiar virtue of this substance is that it can be ignited at a comrade's pipe, and can then be used to set light to the owner's tobacco. Some of the men discovered that the inhalation of the fumes of this burning cordite had a narcotic effect. From this to the ingestion of cordite by the mouth, either solid or dissolved in tea, was only a step. In those unaccustomed to the use of the drug even a small dose causes most unpleasant symptoms, chiefly a very severe racking headache. But the *habitué* can swallow the entire contents of one cartridge. His face flushes, his head throbs and seems to swell, and after a quarter of an hour or so he falls into a deep sleep. On awaking there is thirst and headache. Taken in hot tea, the effect is delirium, followed by sleep. Morphia, opium, and alcohol in small quantities are "pick-me-ups" after cordite eating; and inversely some men used cordite as a reviver after alcoholic excess. Optical and mental delusions, timidity, weakness, and general breakdown, moral and physical, result from long use of cordite as a drug.

### The Diagnosis of Tuberculosis.

The Wassermann test is, as bacteriologists know, simply the adaptation to a particular infective disease, syphilis, of the general principle of what is called the Bordet-Gengou reaction. Captain V. B. Nesfield, of the Indian Medical Service, publishes in the *Lancet* his method of applying the same principle to tuberculosis. For the details of his technique the original article should be consulted. The test is not conducted entirely on the lines of the Wassermann process; indeed, it has the advantage that an immunity index can be got by repeating it with varying quantities of fresh and heated serum respectively. In a doubtful case, says the author, four readings should be taken: before exercise, after exercise, one day after exercise, and two days after exercise. Exercise throws into the circulation an excess of toxin; this reduces the immunity, and hæmolysis may then occur when otherwise it does not. Later there is reaction, which increases the immunity, and hæmolysis no longer occurs. Captain Nesfield has so far tried his test on twenty cases of tuberculosis and ten normal people. So far it has never failed; that is, it has always given a positive reaction in tuberculosis and never in a non-tuberculous case. Admitting that his experience is yet too limited to say definitely what degree of reliance can be placed on the test, the author is still to be credited with a very thoughtful and stimulating piece of work.

### Respiratory Gymnastics for Delicate Children

Dr. Gabriel Simon, in an article in *Paris Médicale* on respiratory gymnastics for children predisposed to tuberculosis, quotes the exercises recommended by Dr. Guinon as suitable for most cases. These are as follows: (1) During inspiration the arms are moved from the sides up in front of the body until they are placed vertically above the shoulders, the fingers of each thus describing a semi-circle in front of the body; during expiration they are allowed to fall to the sides again, but without passing in front of the body, the fingers of each thus describing a semi-circle in a plane at right angles to the first. (2) The forearms are flexed on the arms and the limbs placed horizontally at the shoulder level, the tips of the fingers being in front of the sternum. During inspiration the forearms are abducted and extended horizontally, while during expiration exactly the reverse movements are performed. (3) Rotation of the shoulders from before backwards and from below upwards, with inspiration when the shoulders are in front and above, and with expiration when they are behind and below. This movement is a sort of shrugging of the shoulders round a transverse axis. (4) With the hands touching the thighs, forced abduction and supination of the arms, the hands remaining down, and carried as far back as possible; then adduction and pronation, the hands being brought back to the thighs. Inspiration takes place during the abduction and supination, expiration during the return movements. All the four movements should be carried out in the open, and should be regular, full, and slow (fifteen to twenty per minute). Nasal breathing should be insisted upon. Fatigue should be avoided, and at first the movements should not be carried out for more than ten minutes, but after a while they may be extended to a daily *séance* of half-an-hour's duration. In weaklings it may be necessary to carry out the exercises in a lying-down position. The simplicity of this form of treatment is such that after a preliminary demonstration by the medical man it can safely be left in the hands of the mother or governess, the doctor merely assisting from time to time.

### Urotropine in Meningitis.

In 1908 Casting and Crowe showed that urotropine, even when given by the mouth, was excreted into the cerebro-spinal fluid and was there possessed of antiseptic properties. They argued from this that the drug ought to be used in dealing with cases of meningitis, and also as a prophylactic before operations on the brain and its envelopes. The observations of these American observers have been recently supported by Dr. Ibrahim, who publishes his results in the *Med. Klin.* He has used the drug with success in cases of infantile meningitis, and has always succeeded in recovering it from the cerebro-spinal fluid after ingestion. After a certain time in the body at a temperature of 37° to 38° C. this urotropine dissolved in the fluid is decomposed into formaldehyde. It exercises a definite germicidal action in the cerebro-spinal surroundings, and this observer thinks it justifiable to use it in all cases of serous and suppurative meningitis in young children. He has employed it in daily doses of 8 to 20 grains in infants and up to 40 grains given in five doses of 8 grains each in the case of older children, always without the least sign of dangerous effects.

### A New Occupational Disease.

Professor Gilbert, in *Paris Médicale*, quotes the case of a workman employed in attending to the furnaces of a system of heating by hot-air, who during the past four years has had five attacks of jaundice, accompanied, at any rate so far as the last is concerned, by hepatomegaly and splenomegaly. The individual concerned attributes these attacks to the nature of his employment, and states that on the appearance of the cold weather, after he has been at his work for a few days and has got the furnaces into working order, his icterus commences, and that it disappears and reappears according as he is absent from his work or is once more on duty. The author wishes to know if any other observer has noted cases of a similar nature to the one he describes.

### The Parasite of Infantile Splenic Anæmia.

Dr. Rocco Jemma, of the University of Palermo, claims to have discovered the causal organism of infantile splenic anæmia. This he maintains to be a parasite in every way comparable to that of kala-azar. It is found in the spleen, and it is necessary when searching for it to make use of a trochar in performing splenic puncture, so that a small quantity of the pulp of the organ can be withdrawn for examination. In seven cases recently studied by this method the author has found corpuscles, ovoid or rounded, 3 to 4 $\mu$ in size, some with a central or eccentric nucleus and chromatin granules colouring well with Giemsa's stain. As does kala-azar, splenic anæmia causes an irregular fever of chronic type, splenic and sometimes hepatic hypertrophy, wasting, anæmia, and a high mortality. Unlike the former malady, however, splenic anæmia has no epidemic nor contagious characteristics, and, at any rate in Italy, is almost confined to infants. It may also take an apyretic form, may persist for a long time, and finally end in a cure. From the microbiological standpoint there would seem to be a complete analogy between the organisms of the two diseases, although that of kala-azar cannot be inoculated into the dog, whereas that animal has been inoculated successfully with the organism of splenic anæmia. The identity of the two maladies is not, therefore, completely established. It is nevertheless possible as a result of these researches to diagnose between splenic anæmia with Leishman's parasites and the spleno-megalies with anæmia secondary to syphilis, malaria, tuberculosis, etc.

### Industrial Residues as Cattle-foods.

The disadvantages and dangers of feeding milch-cows on industrial residues are summed up by Dr. Aviragnet in the *Arch. de Med. des Enfants.* Therein he has collected information from the work of various observers, from which he arrives at important conclusions. Various kinds of residues are made use of to feed cattle. Such are (1) those derived from sugar refineries and beet distilleries, called *pulps*; (2) those from distilleries of grain, potatoes, and juniper, known as *distillery-malt*; (3) those from breweries, called *brewery-malt*; and (4) those from oil refineries, known as *oil-cake*. The toxicity of the residues depends upon the poisons of all sorts which are formed by the fermentation and putrefactive processes to which they are submitted before being given to the cattle. These poisons are eliminated in the milk, as can be proved by clinical experimentation, though not by chemical analysis. The author arrives at the following conclusions on this point: (*a*) Beet pulps should be eliminated from the dietary of cows. They are at times harmful when fresh, and after being kept are positively dangerous. Only desiccated pulps can be recommended. (*b*) Distillery-malt is harmful, and should never be used as food. (*c*) Brewery-malt is a good food when fresh, but not when old. (*d*) Foreign oil-cake is usually bad; only native cake should be used. These conclusions only apply to commercial milk. When milk is intended for the use of infants and invalids it should never be procured from cows fed on industrial residues, and regulations to that effect should be instituted, as is already the case in certain countries, such as America and Germany.

